# Down-regulation of miR-let-7e attenuates LPS-induced acute lung injury in mice via inhibiting pulmonary inflammation by targeting SCOS1/NF-κB pathway

**DOI:** 10.1042/BSR20201089

**Published:** 2021-01-04

**Authors:** Wuquan Li, Wentao Zhang, Jun Liu, Yalong Han, He Jiang, Gang Ji, Wenjun Liu

**Affiliations:** 1 Burn Center of Yunnan Province, Second Affiliated Hospital of Kunming Medical University, Kunming, China; 2 Department of Urology Surgery, the Joint Service Support Force 920th Hospital of Chinese PLA, Kunming, China

**Keywords:** Acute lung injury, inflammatory response, let-7e, NF-κB pathway, SOCS1

## Abstract

Excessive pulmonary inflammatory response is critical in the development of acute lung injury (ALI). Previously, microRNAs (miRNAs) have been recognized as an important regulator of inflammation in various diseases. However, the effects and mechanisms of miRNAs on inflammatory response in ALI remain unclear. Herein, we tried to screen miRNAs in the processes of ALI and elucidate the potential mechanism. Using a microarray assay, microRNA let-7e (let-7e) was chose as our target for its reported suppressive roles in several inflammatory diseases. Down-regulation of let-7e by antagomiR-let-7e injection attenuated LPS-induced acute lung injury. We also found that antagomiR-let-7e could obviously improve the survival rate in ALI mice. Moreover, antagomiR-let-7e treatment reduced the production of proinflammatory cytokines (i.e., TNF-α, IL-1β and IL-6) in bronchoalveolar lavage fluid (BALF) of LPS-induced ALI mice. Luciferase reporter assays confirmed that suppressor of cytokine signaling 1 (SOCS1), a powerful attenuator of nuclear factor kappa B (NF-κB) signaling pathway, was directly targeted and suppressed by let-7e in RAW264.7 cells. In addition, it was further observed that SOCS1 was down-regulated, and inversely correlated with let-7e expression levels in lung tissues of ALI mice. Finally, down-regulation of let-7e suppressed the activation of NF-κB pathway, as evidenced by the reduction of p-IκBα, and nuclear p-p65 expressions in ALI mice. Collectively, our findings indicate that let-7e antagomir protects mice against LPS-induced lung injury via repressing the pulmonary inflammation though regulation of SOCS1/NF-κB pathway, and let-7e may act as a potential therapeutic target for ALI.

## Introduction

Acute lung injury (ALI) is a serious inflammatory lung disease with the high mortality (35–40%), which usually caused by various direct or indirect factors, including the trauma, blood transfusion, and infection [1]. In spite of considerable effort, there are still no effective therapeutic regimens for this disease [2]. Therefore, exploring the pathophysiological mechanisms of ALI has important theoretical significance for the prevention and treatment of ALI.

Excessive inflammatory reaction in the lung tissues is the main pathological feature of ALI [3]. Once toll-like receptors (TLRs) are activated, it can subsequently activate nuclear factor-kappaB (NF-κB), induce the transcriptional expressions and release of massive inflammatory mediators, and promote the cascade amplification of inflammation, and form a ‘waterfall’ reaction of inflammation in the lung, leading to the occurrence of ALI [4,5]. Pan et al. showed that Interleukin-35 (IL-35) improved acute lung injury through suppressing inflammatory response by blocking the activation of NF-κB pathway in a LPS-induced mice ALI model [6]. Zhang et al. reported that L-lysine ameliorated sepsis-induced ALI in a LPS-induced mouse model via inhibition of inflammatory response [7]. Therefore, the inhibition of the excessive inflammatory response may be an effective therapeutic regimen for ALI.

MicroRNAs (miRNAs) are a family of short, small, noncoding RNAs (an average size of 22 nucleotides), which suppresses target gene expression through either translation repression or RNA degradation [8,9]. Numerous studies have reported that miRNAs is involved in the regulation of pulmonary inflammatory diseases [10,11]. In ALI mice model, a large deal of miRNAs was found to be differently expressed, which are accompanied by the alterations in inflammatory factors, indicating that miRNAs may play important roles in the regulation of pulmonary inflammation in ALI [12]. Yan et al. demonstrated that miR-223 attenuated LPS-induced lung inflammation in mice via the NLRP3 inflammasome and TLR4/NF-κB signaling pathway [13]. Yang et al. found that miR-140-5p up-regulation inhibited the production of inflammatory cytokines in ALI by targeting TLR4 [14]. Therefore, more miRNAs and their roles involved in the inflammatory response in ALI need further studies.

In the present study, we established an *in vivo* mice model of ALI to analyze the microRNAs expression patterns, and the roles and underlying mechanisms of let-7e in the inflammatory response in ALI were further examined. We found that down-regulation of let-7e suppressed the NF-κB pathway activation by targeting SOCS1, leading to the improvement of lung injury in mice. Our results suggest that let-7e may act as a promising novel therapy target for the prevention and treatment of ALI.

## Materials and methods

### Animals and treatment

Male BALB/c mice (18–22 g) at 6 weeks old were obtained from Shanghai SLAC Laboratory Animal Co., Ltd. BALB/c mice were housed under standard conditions (12-h light–dark cycle, 25-27°C, ∼40% humidity) with free access to food and water throughout the duration of the experiments. All animal studies were performed according to the guidelines of the Chinese Council on Animal Care and ethical approval was obtained for the use of animals prior to the start of the study from the ethics committee of the Second Affiliated Hospital of Kunming Medical University (Approval number: 2018-00113). All animal works were done in the Experimental Animal Center of Kunming Medical University.

All mice were randomly divided into four groups (*n*=10/group): (1) Control group, (2) LPS/ALI group, (3) LPS + antagomir-let-7e group, (4) LPS + antagomir-negative control (NC) group. LPS/ALI group mice were injected intravenously (tail vein) with 0.2 mL LPS (10 mg/kg). The control group received the normal saline (NS). Mice in antagomir-let-7e or antagomir NC group were injected intravenously (tail vein) with 0.2 ml antgomir-let 7e or antagomir NC (8 mg/kg [15]) 24 h prior to LPS. Antgomir-let 7e (5′-ACTATACAACCTCCTACCTC-3′) and antagomir negative control (NC) (5′-CAGUACUUUUGUGUAGUACAA-3′) were synthesized by GenePharma Co., Ltd. Except survival experiment, all mice were anesthetized by an intraperitoneal injection of pentobarbital sodium (50 mg/kg) followed by cervical dislocation, and mortality was confirmed by observation [16]. After killing, the lung was excised as an intact unit. With 1 ml sterile ice-cold PBS, the trachea was lavaged to collect the bronchoalveolar lavage (BAL) fluid. The BALF fluid was pooled and centrifuged at 400 × ***g*** for 10 min at 4°C to pellet the cell fraction and the supernatant was stored at −80°C until mediator analysis. The survival rate was observed from 0 to 7 days using the Kaplan–Meier methods.

### Microarray

Total RNA were extracted from lung tissues of ALI mice and control mice (*n*=3) using miRNeasy mini kit (Qiagen, West Sussex, U.K.) according to the manufacturer’s protocol. The samples were assessed using the miRCURY™ LNA Array (v.18.0). The procedure and imaging processes were as described previously [17]. In order to validate the expression levels obtained in the microarray experiments, we used quantitative real-time RT-PCR (qRT-PCR).

### qRT-PCR analysis

Total RNA was isolated from lung tissues or cells using RNeasy Mini Kit (Qiagen Corporation, Hilden, Germany). Reverse transcription of let-7e and SOCS1 (1 µg) was synthesized using the MicroRNA Reverse Transcription Kit (Thermo Fisher Scientific, Inc) and the PrimeScript RT Reagent Kit (Takara Bio, Inc., Tokyo, Japan), respectively. Let-7e and SOCS1 expression were measured using the SYBR Green mixture (Solarbio, Beijing, China) on a Light Cycler instrument (Bio-Rad). The primers for qRT-PCR analysis were as follows: let-7e forward, 5′-AGCAAGCTTTGGCACCCACCCGTAGAAC-3′ and reverse, 5′-TAAGGATCCGATGCAGGGACAAGGACAGAA-3′; U6 forward, 5′-CTCGCTTCGGCAGCACA-3′ and reverse, 5′-AACGCTTCACGAATTTGCGT-3′. SOCS1 forward, 5′-CTTCTGTAGGATGGTAGCACAC-3′, 5′-AGGAAGAGGAGGAAGGTTCT-3′; GAPDH R: 5´-GTGGTGAAGACGCCAGTGGA-3´; F: 5´-CGAGCCACATCGCTCAGACA-3´. The procedures of amplification reaction were performed as follows: 5 min at 95°C, followed by 40 cycles of 95°C for 30 s and 60°C for 45 s, 30 min at 72°C. The miRNA relative expressions were analyzed using the 2^−ΔΔCT^ method [18].

### Lung histology

Upper and lower lobe lung samples were excised 24 h after LPS challenge, fixed with 10% formalin and microsectioned at 5 μm. Then, the tissues embedded in paraffin and stained with haematoxylin and eosin (HE). Lung injury score was evaluated in a blinded fashion as previously described [19]. The pictures were captured by a microscope (Nikon E-800M, Tokyo, Japan) at ×400 magnification.

### Evaluation of lung permeability

Evans blue (EB) dye extravasation method was used to assess pulmonary permeability as previously described [20]. In a brief, 4 ml/kg of 2% Evans blue (Sigma-Aldrich, St. Louis, MO, U.S.A.) in normal saline in normal saline was injected into mice of each group through the tail vein. After dye injection 2 h, mice were killed and then the dye was extracted by incubation in formamide for 24 h at 60°C. The light absorbance at 620 nm was measured and the dye concentration was reported as the ratio of absorbance relative to the amount of dry lung tissue (μg/100 mg dry tissues).

### Lung wet/dry (W/D) ratio

The W/D ratio was used to calculate pulmonary edema. After 24 h LPS challenge, mice were sacrificed, the right lungs was taken and immediately weighted, then dried in an incubator at 55°C for 60 h.

### Oxygenation index (PaO_2_/FiO_2_) analysis

Twenty-four hours after ALI (or control group), mice were anesthetized 3% chloral hydrate and endotracheal intubation was performed with a 20-gauge catheter. Pure oxygen was mechanically ventilated at 7 ml/kg (120 times/min). After 20 min of ventilation, the carotid blood was collected and measured with a commercial blood gas analyzer (ABL 8000; radiometer Copenhagen, West Lake, Ohio).

### Measurement of proinflammatory cytokines

The IL-6, IL-1β and TNF-α levels in BALF were analyzed by using IL-6 (cat no.p1330), IL-8 (cat no.p1640) and TNF-α (cat no.pt518) ELISA Kits according to the kit instructions. All kits were obtained from Beyotime Biotechnology, Shanghai, China.

### Cell culture and transfection

RAW264.7 cells were obtained from Sciencell Research Laboratories (Carlsbad, CA, U.S.A.). RAW264.7 cells were maintained in DMEM supplemented with 10% FBS (Sigma-Aldrich), 1% penicillin and streptomycin (Sigma-Aldrich) at 37°C and 5% CO_2_ incubator.

When RAW264.7 cells grown to about 80% confluence in six-well plate, 20 nM let-7e mimics and let-7e inhibitor were transfected into cells at 37°C for 48 h using Lipofectamine® 2000 (Invitrogen). The let-7e mimics, mimics negative control (NC), let-7e inhibitor and inhibitor NC were obtained from RiBoBio (Guangzhou, China). let-7e mimics (UGAGGUAGUAGAUUGUAUAGUU); mimics-NC (5′-UGAGGUAGUAGAUUGUAUAGUU-3′); let-7e inhibitor (5′-ACGUGACACGUUCGGAGAATT-3′); inhibitor NC (5′-ACGUGACACGUUCGGAGAATT-3′)

### Luciferase assay

The 3´-UTR of SOCS1, with wild-type or mutant (Mut) binding sites for let-7e, was amplified and cloned into the pGL3 vector (Promega, Madison, WI, U.S.A.) to generate the plasmid pGL3-WT-SOCS1-3´-UTR or pGL3-Mut-SOCS1-3´-UTR, respectively. The dual-luciferase reporter assay was performed as described previously [21]. RAW264.7 cells were treated with let-7e mimics, Let-7e inhibitor and the luciferase reporter plasmids, pRL-TK-Renilla control plasmid (Promega) using Lipofectamine 2000 (Invitrogen). At 48 h post-transfection, luciferase activities were detected using the Dual Luciferase Reporter Kit (Beyotime Biotechnology, Jiangsu, China).

### Western blot

Western blot was performed as previously described [21,22]. Briefly, total protein was extracted using radio immune-precipitation assay (RIPA) buffer and the protein concentration determined using the bicinchoninic acid (BCA) assay (Pierce; Thermo Fisher Scientific, Inc.). The extraction and isolation of nuclear and cytoplasmic proteins were performed according to the Cytoplasmic and Nuclear Protein Extraction Kit (cat no. 78833, ThermoFisher Scientific, U.S.A.). About 40 μg extracted proteins were separated by 12% SDS-PAGE (w/v) and transferred onto a PVDF membrane (Millipore). The membranes were blocked with 1% BSA for 2 h at room temperature, followed by incubation with several primary antibodies against SOCS1 (cat no.14358, 1:2000), nuclear p-p65 (cat no.3033, 1:1,000 dilution), p-IκB-α (cat no.2859, 1:1000 dilution), IκB-α (cat no.4814, 1:1000 dilution) and β-actin (ab8226, 1:1000) (all from Cell Signaling Technology, Inc., Danvers, MA, U.S.A.) at 4°C overnight, followed by HRP-conjugated goat anti-rabbit IgG (1:10,000; cat. no. 205718; Abcam, Cambridge). The protein bands were developed using ECL kit (GE Healthcare) and blot bands were quantified with ImageJ version 1.46 (Rawak Software, Inc. Munich, Germany).

### Statistical analysis

GraphPad Prism 5.0 (La Jolla, CA, U.S.A.) was used to analyze the statistical analyses. Statistical differences were analyzed using the Student’s *t-*test or one-way analysis of variance (ANOVA) with the Tukey’s test. All data are presented as the mean ± SD; *P*<0.05 was defined as significant.

## Results

### let-7e was up-regulated in lung tissues and BALF in ALI mice

**Figure 1:**
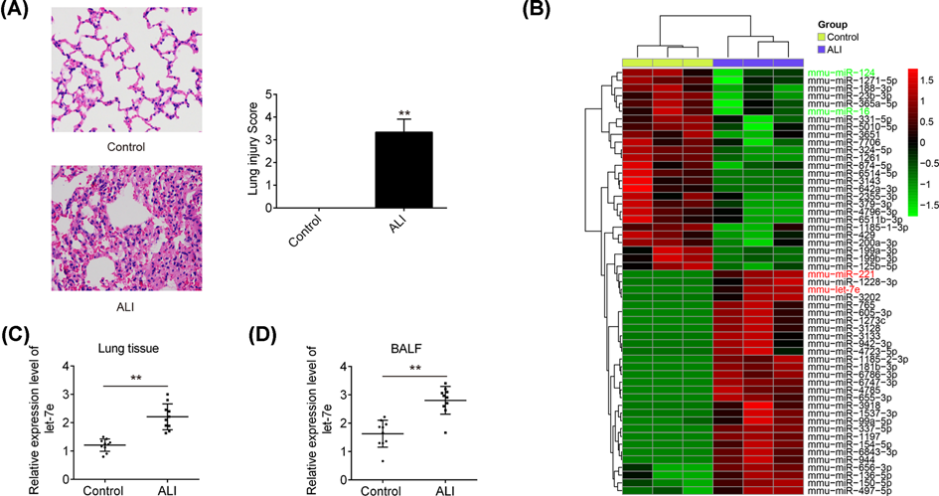
Let-7e was up-regulated in LPS-induced ALI in mice Mice (*n*=3 each) were treated with a single dose of LPS (2 mg/kg) or normal saline and total RNAs were isolated from their lungs at 24 h after LPS treatment. (**A**) H&E-stained sections of lung tissue from control and LPS treated mice (400 × magnification). (**B**) Heat map of miRNA profiles represented the significantly regulated miRNAs (*n*=3/group). (**C** and** D**) miR-221 expression was validated by qRT-PCR in lung tissues and BALF of mice challenged with LPS (*n*=10/group). Data represent the mean ± SD of three independent experiments. **P<0.01 vs. control group.

First, an experimental mouse model of ALI induced by LPS was established as previously described [13], and then the histopathological analysis of mice were evaluated. As shown in Figure 1A, increased inflammatory cell infiltration in the alveolar space, as well as the swelling of types I and II alveolar epithelial cell and rupturing of alveolar basement membrane were observed in ALI group compared with control group. Using a lung-injury score system, higher scores were found in ALI group than that in the control group, suggesting that ALI model induced by LPS was successfully established in mice.

To identify miRNAs involved in the inflammation of ALI, a miRNA microarray was performed in ALI mice. As shown in Figure 1B, it was found that 28 up-regulated and 26 down-regulated miRNAs. Among these aberrant miRNAs, miR-124 and miR-16 were decreased, and miR-221 was increased, which are consistent with previous reports [17,23–25], indicating the reliability of our microarray. Let-7e was chose for subsequent investigation due to its important role in regulation of inflammatory response in various diseases [26–28]. However, the underlying mechanisms of let-7e in the inflammatory reactions of ALI remain unclear until now.

To verify the results of above miRNA microarray, let-7e expression was measured in lung tissues and BALF from ALI mice by qRT-PCR. The results showed that let-7e was much higher in lung tissues and BALF in ALI mice than that in controls (Figure 1C,D). All these data suggest that let-7e may be involved in the pathogenesis of ALI.

### Down-regulation of let-7e improves LPS-induced lung injury in mice

**Figure 2:**
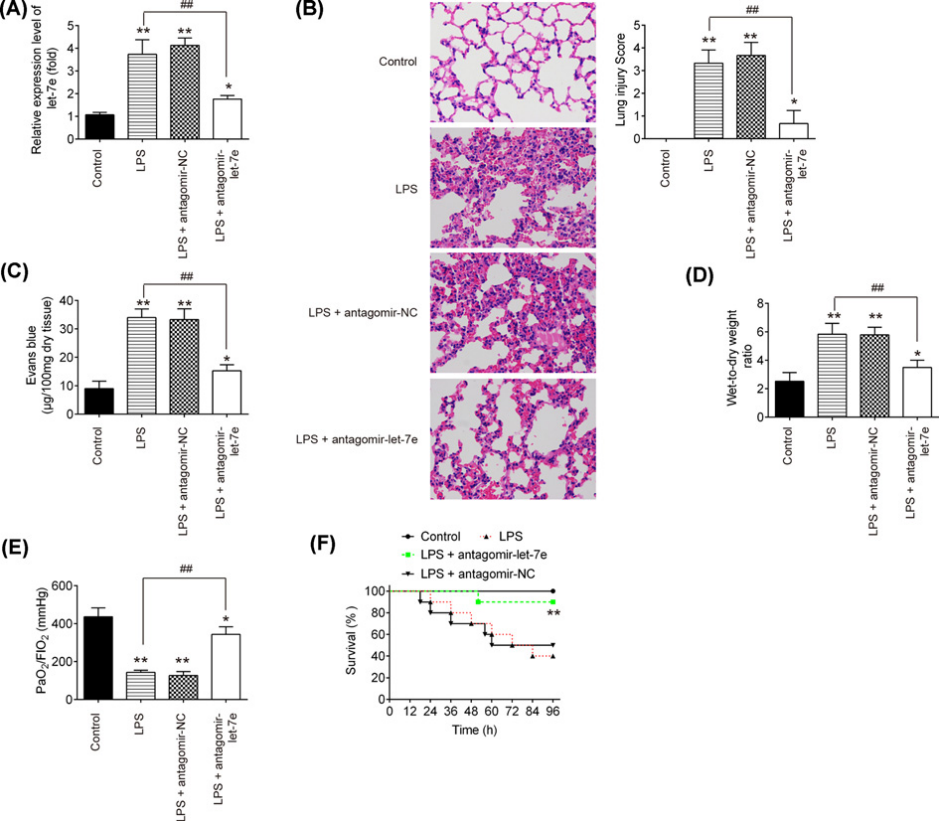
Let-7e knockdown improved LPS-induced injury in mice Mice were injected intravenously (tail vein) with 8 mg/kg antagomir-let-7e or antagomir-NC 24 h prior to 2 mg/kg LPS treatment. The mice were killed after LPS administration for 24 h and then lung tissues were collected for analysis. (**A**) The expression of let-7e was determined by qRT-PCR. (**B**) The degree of injury of lung samples was assessed with H&E staining at ×400 magnification (*n*=10/group). (**C**) Lung permeability was assessed using the Evans blue dye extravasation method (*n*=10/group). (**D**) The pulmonary edema was evaluated by the lung wet/dry weight ratio (*n*=10/group). (**E**) PaO_2_/FiO_2_ was measured using a commercial blood gas analyzer. (**F**) The Kaplan–Meier method was used to compare the survival rates among different groups (*n*=10/group). *P<0.05, **P<0.01 vs. control group. ##*P*<0.01 vs. LPS alone group.

To examine the influences of let-7e in ALI mice, antagomir-let-7e or antagomir NC (8 mg/kg) were intravenously injected into mice, followed by LPS treatment. Twenty-four hours after LPS challenge, BALF and lung samples were collected for analyses. As shown in Figure 2A, let-7e was significantly up-regulated in response to LPS treatment. However, the expression of let-7e was obviously reduced in antgomir-let-7e plus LPS group, compared with LPS group. HE staining showed that antgomir-let-7e injection significantly alleviated the lung injury, along with lower lung injury score, compared with LPS group (Figure 2B). Evans Blue assay was performed to evaluate the capillary permeability. It was observed that LPS increased the capillary permeability compared with control group, whereas antagomir-let-7e dramatically reduced the capillary permeability compared with LPS group (Figure 2C). Moreover, the lung W/D ratio was also used to assess the extent of lung edema and it was found that the lung W/D ratio of mice in the LPS group was higher than that of controls, whereas antgomir-let-7e injection obviously reduced the lung W/D ratio in ALI mice (Figure 2D). At present, PaO_2_/FiO_2_ ratio has become a vital indicator for pulmonary function [29]. Therefore, we also assessed the influence of let-7e in PaO2/FiO2 ratio, and the results showed that PaO_2_/FiO_2_ was markedly decreased in LPS group, but increased by antgomir-let-7e (Figure 2E). Finally, the survival rates of mice were examined in these groups. The survival rate of mice in LPS group was much lower than that in control group, while antagomir-let-7e obviously improved the survival rate compared with that in LPS group (Figure 2F). All data suggest that down-regulation of let-7e protected mice against LPS-induced lung jury.

### Down-regulation of let-7e inhibited LPS induced inflammation in mice

**Figure 3:**
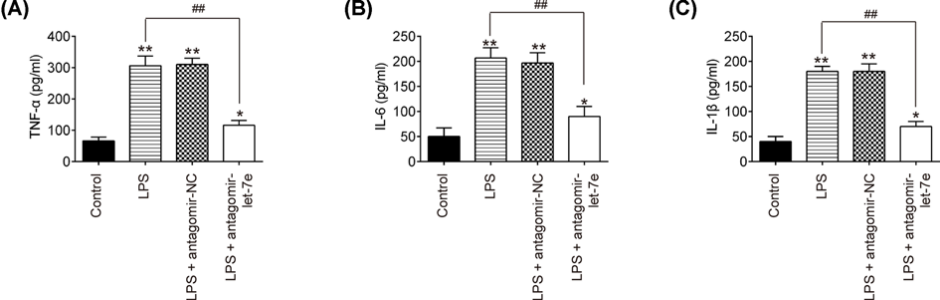
Let-7e knockdown suppressed LPS-induced inflammation in mice Mice were injected intravenously (tail vein) with antagomir-let-7e, antagomir-NC (8 mg/kg) at 24 h prior to 2 mg/kg LPS treatment. The mice were killed after LPS administration for 24 h and then BALF were collected for analysis. (**A–C**) TNF-α, IL-6 and IL-1β levels in BALF were measured using commercial ELISA kits (*n*=10/group). Data represent the mean ± SD of three independent experiments; *P<0.05, **P<0.01 vs. control group. ##*P*<0.01 vs. LPS alone group.

It is well-known that excessive inflammatory response is critical in the development of ALI. To examine whether let-7e affects the inflammatory response in ALI, the secretion of many crucial proinflammatory cytokines including TNF-α, IL-6 and IL-1β was measured in BALF of ALI mice. It was observed that LPS treatment could up-regulate these cytokines secretion, whereas the promoting effects of LPS were attenuated by antgomir-let-7e (Figure 3A–C). These results reflect the fact that down-regulation of let-7e inhibited LPS induced inflammation in mice.

### SOCS1 is a direct target of let-7e

**Figure 4:**
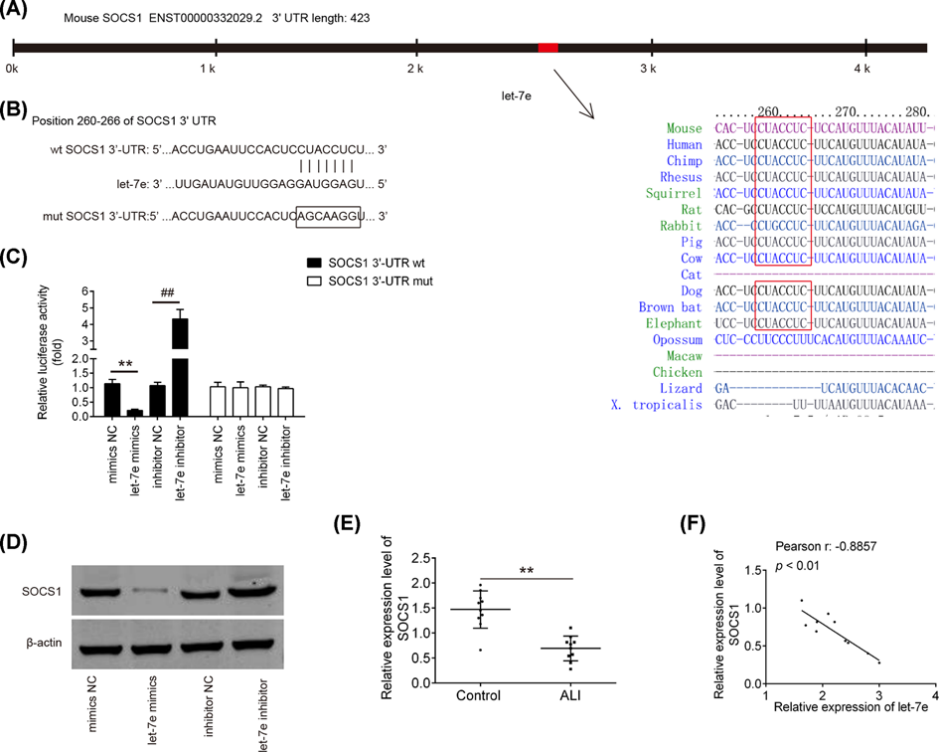
SOCS1 was a direct target of let-7e (**A** and** B**) The putative binding site of let-7e and SOCS1 is shown. (**C**) Luciferase assay of RAW264.7 cells co-transfected with firefly luciferase constructs containing the SCOS1 wild-type or mutated 3´-UTRs and let-7e mimics, mimics NC, let-7e inhibitor or inhibitor NC, as indicated (*n*=3). Data represent the mean ± SD of three independent experiments. **P<0.01 vs. mimics NC, ## *P*<0.01 vs. inhibitor NC. (**D**) The expressions of SOCS1 protein after transfection with let-7e mimics or let-7e inhibitor were measured by Western Blot. (**E**) Expression of SOCS1 in lung tissues was measured using qRT-PCR in ALI mice (*n*=10/group). **P<0.01 vs. Blank group. (**F**) Spearman’s correlation was used to assess the correlation of let-7e and SOCS1 expression in lung tissues from ALI mice (*r* : -0.8857; *P*<0.01).

To explore the molecular mechanism responsible for inflammation suppression induced by let-7e in ALI mice, TargetScan and PicTar databases were used to predict the targets of let-7e, especially for those that are associated with inflammation. SOCS1, a powerful attenuator of NF-κB pathway, was identified as a downstream target of let-7e (Figure 4A,B). To validate whether SOCS1 was directly targeted by let-7e, a luciferase reporter assay was conducted. The results showed that the overexpression of let-7e significantly inhibited the luciferase activity of the SOCS1-3´UTR wt, while let-7e inhibition promoted the luciferase activity. However, no significant change was observed in luciferase activity of SOCS1-3´UTR mut (Figure 4C). Western Blot results revealed that let-7e mimics markedly down-regulated the expression of SOCS1 protein, whereas let-7e inhibitor promoted the expression of SOCS1 in RAW264.7 cells (Figure 4D). Additionally, the expression level of SOCS1 in the lung tissues from ALI mice was detected by qRT-PCR. As shown in Figure 4E, SOCS1 was significantly down-regulated in LPS group, compared with that in control group. Moreover, an inverse correlation was observed between SOCS1 and let-7e in lung tissues of ALI mice (Figure 4F). These data suggest that SOCS1 is a functional target of let-7e.

### let-7e blocked the activation of SOCS1/NF-κB signaling pathway

**Figure 5:**
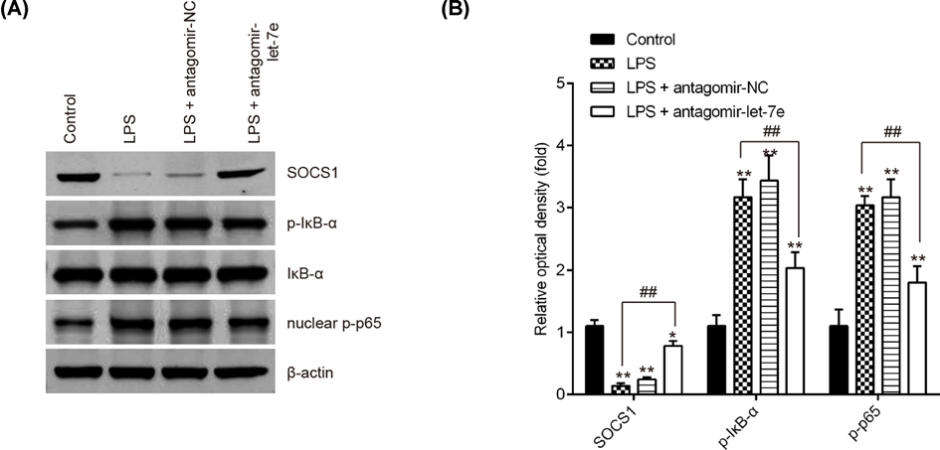
Let-7e blocked the activation of SOCS1/NF-κB pathway in LPS-induced ALI mice Mice were injected intravenously (tail vein) with antagomir-let-7e, antagomir-NC (8 mg/kg) 24 h prior to 2 mg/kg LPS. The mice were sacrificed at 24 h after LPS administration and then lung tissues were collected for analysis. (**A**) The levels of SOCS1, IκB-α, p-IκB-α and nuclear p-p65 were measured by Western Blot (*n*=3). (**B**) The bands were semi-quantitatively analyzed by using ImageJ software, normalized to β-actin density. Data represent the mean ± SD of three independent experiments. *P<0.05, **P<0.01 vs. control group. ##*P*<0.01 vs. LPS alone group.

**Figure 6:**
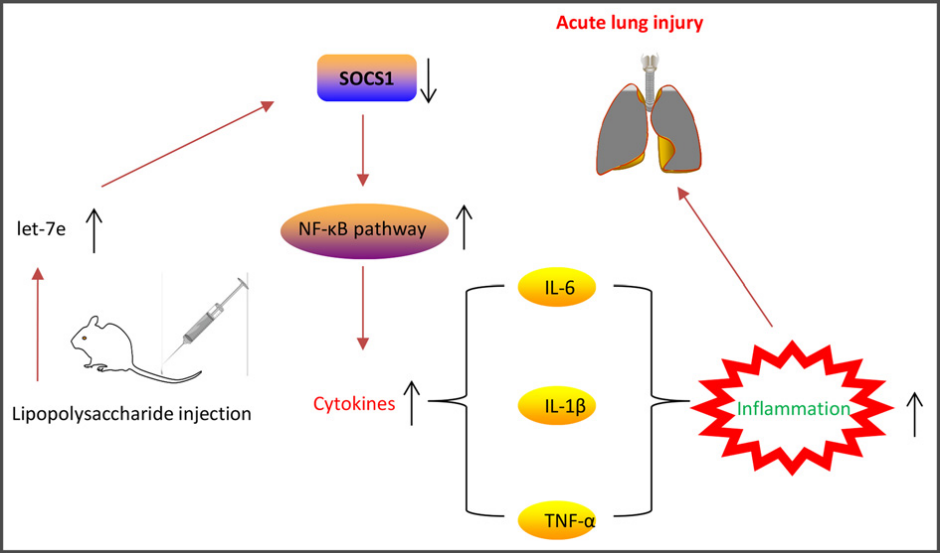
Schematic diagram of the signaling pathway in which let-7e overexpression promotes LPS-induced lung injury in mice Let-7e was highly expressed after LPS-stimulated in mice, then it used the inhibitory machinery to reduce the expression of its target gene SOCS1, thereby promoting the NF-κB pathway activation and inducing cytokine production (for example IL-6), resulting in lung injury.

Previous studies have shown that the activation of the NF-κB signaling pathway results in the release of proinflammatory cytokines and accelerated the development of ALI [30,31]. Considering that let-7e directly targeted SOCS1, we hypothesized that SOCS1/NF-κB signaling pathway may be involved in the protective effects of let-7e in ALI mice. To test this hypothesis, the expression levels of NF-κB pathway-related core factors, nuclear p-p65 and p-IκB-α were determined by Western Blot. As shown in Figure 5A,B, LPS treatment significantly up-regulated the expression of p-p65 and p-IκB-α, and down-regulated the expression of SOCS1, suggesting that LPS treatment lead to the activation of NF-κB pathway. But, antgomir-let-7e blocked the activated NF-κB pathway caused by LPS, as evidenced by the reduction of p-p65 and p-IκB-α expressions, and promotion of SOCS1 expression. These data suggest that let-7e knockdown may suppress the inflammation through blocking the activation of SOCS1/NF-κB pathway in ALI mice (Figure 6).

## Discussion

In the present study, we found let-7e was highly expressed in lung tissues and BALF in ALI mice. Down-regulation of let-7e improved lung injury caused by LPS through suppressing the inflammatory response in mice. Moreover, suppression of SOCS1/NF-κB signaling pathway may contribute to the protective effect of let-7e antagomir in ALI mice. All these data suggest that knockdown of let-7e may act as a potential therapeutic approach for the treatment of ALI.

Numerous evidences have reported that miRNAs are implicated in the pathogenesis of ALI. For instance, Yang et al. discovered that overexpression of miR-142a-3p alleviated LPS-induced lung injury through inhibiting inflammatory response [32]. Pan et al. showed that miR-124 improved the septic shock induced lung injury in mice through inhibiting the MAPK signaling pathway [23]. Yu et al. found that overexpression of miR-145-5p exerted as a protective role in LPS-induced ALI [33]. Yang et al. reported that miR-16 had a protective effect against ALI via attenuating inflammatory response in mice [25]. Huang et al. demonstrated that miR-27b inhibition alleviated LPS-induced ALI via activation of NF-E2-related factor 2 (Nrf2) [34]. Fu et al. reported that miR-92a attenuated LPS-induced pulmonary inflammation and injury in mice [35]. In the present study, it was found that let-7e was significantly increased in lung tissues from ALI mice model by a miRNA microarray and its high expression was validated in lung tissues and BALF in ALI mice. All data suggest that let-7e may be involved in the pathophysiological process of ALI.

Recent studies have shown that let-7e play important roles in inflammation-induced diseases. For example, Tang et al. found that let-7e promoted the inflammatory response through PI3K/Akt signaling pathway in spinal cord injury (SCI) mice model [36]. Li et al. have shown that inhibition of the let-7e induced cardioprotection against ischemia–reperfusion injury through Akt and mTOR pathways [37]. Wang et al. reported that knockdown of let-7 exerted a neuroprotective effect following cerebral ischemia–reperfusion injury by up-regulating MKP1 expression, reduced apoptosis and the inflammatory reaction [38]. Of note, Rao et al. demonstrated the role of let-7e in the regulation of inflammatory genes in staphylococcal enterotoxin B-induced acute inflammatory lung injury model [39]. However, the mechanisms underlying the inflammatory response in ALI remain unclear. In our study, we demonstrated that knockdown of let-7e by antagomir-let-7e improved LPS-induced lung injury, as evidenced by the reduction of capillary permeability and the lung W/D ratio, and promotion of PaO_2_/FiO_2_, as well as increased survival rates of mice. Moreover, it was observed that knockdown of let-7e significantly reduced LPS induced secretion of proinflammatory cytokines. All data indicate that let-7e exerted its protective effects against ALI through suppressing inflammation.

SOSC1, a negative regulator of cytokine signaling, has received attention in various inflammatory diseases including ALI because of its ability to suppress inflammatory response [40], in addition to its role in human cancers [41]. For instance, Galam et al. found that SOCS-1 exhibits its protective role against ALI by suppressing proinflammatory cytokines production [42]. Zhang et al. showed that SOCS-1 ameliorated smoke inhalation-induced ALI through inhibition of apoptosis, another major contributor to the pathogenesis of ALI [43]. In our study, SOCS1 was found to be directly targeted by let-7e through bioinformatics analysis and luciferase assay. More importantly, SOCS1 was significantly down-regulated and inversely correlated with let-7e levels in lung tissues from ALI mice. All data suggest that let-7e antagomir may suppress the inflammation through promoting the expression of SOCS1.

It is well-known that SOCS-1 is an upstream regulator of the NF-κB pathway. SOCS-1 can influence NF-κB pathway through binding to the NF-κB-p65 subunit of NF-κB, and then promote the ubiquitination, resulting in degradation of NF-κB-p65 [44,45]. Interestingly, a previous study has demonstrated that let-7e modulated the inflammatory response in vascular endothelial cells through NF-κB pathway [26]. Therefore, we further explored the SOCS-1/NF-κB signaling pathway in relation to let-7e expression. It was found that knockdown of let-7e up-regulated the expression of SOCS-1, down-regulated the expressions of NF-κB pathway-related core factors, nuclear p-p65 and p-IκB-α in ALI mice. These results suggest that let-7e suppressed the inflammatory response through SOCS-1/NF-κB signaling pathway.

In conclusion, we demonstrated that down-regulation of let-7e inhibits LPS-induced inflammatory responses via inhibition of the SOCS1/NF-κB pathway. The let-7e/SOCS1/NF-κB axis may serve as a promising target for the treatment of ALI.

## Data Availability

All the data presented in the present study is available from the corresponding author upon reasonable request.

## Abbreviations

ALI: acute lung injury
BALF: bronchoalveolar lavage fluid
EB: Evans blue
miRNA: microRNA
NF-κB: nuclear factor kappa B
Nrf2: NF-E2-related factor 2
SCI: spinal cord injury
SOCS1: suppressor of cytokine signaling 1
TLR: toll-like receptor
W/D: wet/dry
